# Frailty in COPD: an analysis of prevalence and clinical impact using UK Biobank

**DOI:** 10.1136/bmjresp-2022-001314

**Published:** 2022-07-04

**Authors:** Peter Hanlon, James Lewsey, Jennifer K Quint, Bhautesh D Jani, Barbara I Nicholl, David A McAllister, Frances S Mair

**Affiliations:** 1 Institute of Health and Wellbeing, University of Glasgow, Glasgow, UK; 2 National Heart and Lung Institute, Imperial College London, London, UK

**Keywords:** COPD epidemiology, COPD Exacerbations, Clinical Epidemiology

## Abstract

**Background:**

Frailty, a state of reduced physiological reserve, is common in people with chronic obstructive pulmonary disease (COPD). Frailty can occur at any age; however, the implications in younger people (eg, aged <65 years) with COPD are unclear. We assessed the prevalence of frailty in UK Biobank participants with COPD; explored relationships between frailty and forced expiratory volume in 1 second (FEV1) and quantified the association between frailty and adverse outcomes.

**Methods:**

UK Biobank participants (n=3132, recruited 2006–2010) with COPD aged 40–70 years were analysed comparing two frailty measures (frailty phenotype and frailty index) at baseline. Relationship with FEV1 was assessed for each measure. Outcomes were mortality, major adverse cardiovascular event (MACE), all-cause hospitalisation, hospitalisation with COPD exacerbation and community COPD exacerbation over 8 years of follow-up.

**Results:**

Frailty was common by both definitions (17% frail using frailty phenotype, 28% moderate and 4% severely frail using frailty index). The frailty phenotype, but not the frailty index, was associated with lower FEV1. Frailty phenotype (frail vs robust) was associated with mortality (HR 2.33; 95% CI 1.84 to 2.96), MACE (2.73; 1.66 to 4.49), hospitalisation (incidence rate ratio 3.39; 2.77 to 4.14) hospitalised exacerbation (5.19; 3.80 to 7.09) and community exacerbation (2.15; 1.81 to 2.54), as was frailty index (severe vs robust) (mortality (2.65; 95% CI 1.75 to 4.02), MACE (6.76; 2.68 to 17.04), hospitalisation (3.69; 2.52 to 5.42), hospitalised exacerbation (4.26; 2.37 to 7.68) and community exacerbation (2.39; 1.74 to 3.28)). These relationships were similar before and after adjustment for FEV1.

**Conclusion:**

Frailty, regardless of age or measure, identifies people with COPD at risk of adverse clinical outcomes. Frailty assessment may aid risk stratification and guide-targeted intervention in COPD and should not be limited to people aged >65 years.

**WHAT IS ALREADY KNOWN ON THIS TOPIC**

- Frailty is common in people with chronic obstructive pulmonary disease (COPD), including in younger people (eg, those aged less than 65 years); however, the clinical implications of COPD in this age group are poorly understood.

**WHAT THIS STUDY ADDS**

- Frailty in people with COPD aged 40–70 is associated with increased risk of mortality, hospital admission, major adverse cardiovascular events and COPD exacerbations.
- This relationship is independent of the severity of airflow limitation.

**HOW THIS STUDY MIGHT AFFECT RESEARCH, PRACTICE OR POLICY**

- Current policies for frailty identification tend to focus exclusively on those aged 65 and over. These findings suggest that in people with COPD, identifying frailty in younger people may aid risk stratification an identification of those for whom interventions may be designed and targeted.

## Introduction

Chronic obstructive pulmonary disease (COPD), characterised by fixed and progressive airflow obstruction, is the third leading cause of death worldwide.^1^ COPD is also a condition associated with ageing. While it is estimated that 10% of the adult population worldwide may be living with COPD,^1^ the prevalence increases from <5% in people aged <65 years to >20% in people aged >85 years.^2^ This has highlighted the need to understand the links between COPD and states associated with ageing, such as frailty.^3 4^ However, neither frailty nor COPD exclusively affect older people, and there is no clearly defined threshold above which frailty becomes a clinically meaningful concept. Most studies of frailty have focused exclusively on people over the age of 65, in whom frailty is more common. Frailty can affect people across a range of ages,^5 6^ including people aged <65 years in whom it has been far less frequently studied. The clinical implications of frailty at younger ages remain unclear.

Frailty describes a state of reduced physiological reserve.^7^ People living with frailty are more vulnerable to decompensation and adverse health outcomes in response to physiological stress. This confers an increased risk of a range of outcomes including mortality, hospital admission, adverse drug reactions and falls.^8^ COPD is associated with a range of extrapulmonary complications including cardiovascular morbidity,^9^ osteoporosis,^10^ and muscle weakness,^11^ all of which may contribute to frailty.

Frailty is highly prevalent in people with COPD.^12^ Most previous studies have focused exclusively on people aged >65 years.^5 13–15^ However, none of these studies have explored the clinical implications of frailty in younger people with COPD. Furthermore, while some studies have demonstrated an association between frailty and both severity of airflow limitation^16–18^ and mortality in people with COPD,^19–21^ these findings have been inconsistent.^22–25^ It is also not clear if the relationship between frailty and adverse outcomes in COPD is independent of the severity of COPD assessed by airflow limitation.

This study seeks to address these gaps using data from the UK Biobank, a cohort of people aged 40–70, representing a relatively younger age range than most previous studies. It will assess two models of frailty; the frailty index and the frailty phenotype. We aim: (1) to assess the prevalence of frailty in UK Biobank participants with COPD, (2) to explore the relationship between frailty and FEV1 and (3) to quantify the association between frailty and mortality, hospitalisations, major adverse cardiovascular events (MACE) and COPD exacerbations.

## Methods

This is an observational analysis of the prevalence and impact of frailty, assessed using two different definitions, in UK Biobank participants with COPD.

### Study population

UK Biobank is a large cohort, recruited by invitation between 2006 and 2010 (5% response rate). Participants were aged between 40 and 70 and had to be registered with a general practitioner and live within 20 miles of one of 22 assessment centres in England, Scotland and Wales. Participants underwent a baseline assessment questionnaire, nurse interview, physical assessment and provided biological samples. Informed consent was also given for linkage to healthcare records including primary care, hospital episode statistics and national mortality records. Currently, linked primary care records are available for 218 570 of the original 502 533 participants. Participants with available primary care data are similar to the wider UK Biobank cohort in terms of age, sex, socioeconomic status and self-reported long-term conditions (online supplemental appendix 1).

10.1136/bmjresp-2022-001314.supp1 Supplementary data

### Identifying COPD

Participants with COPD were identified from linked primary care data using a previously validated list of diagnostic codes (Read-codes).^26^ This code list has been shown to have a high positive predictive value for COPD (86.5%). We included participants with any relevant code occurring prior to UK Biobank baseline assessment. We did not include people with self-reported COPD if they did not have a corresponding primary care Read code.

### Spirometry

We assessed the severity of COPD using spirometry data. We relied primarily on spirometry values coded in primary care records in the 2-year period prior to baseline assessment, as the quality of spirometry undertaken in primary care is known to be high.^27^

Where no primary care measures were available, we used spirometry data from UK Biobank baseline assessment. These measurements were taken using a Vitalograph Pneumotrac 6800 according to American Thoracic Society/European Respiratory Society guidelines. No postbronchodilator measurements were taken. Criteria for acceptable spirometry values from UK Biobank assessment data were taken from previous UK Biobank studies and are described in full in online supplemental appendix 1.^28^

We did not use spirometry to confirm the diagnosis of COPD as UK Biobank spirometry was not postbronchodilator, and previous studies demonstrated that the addition of spirometry only marginally improves the positive predictive value of the diagnostic codes used to identify COPD.

For all analyses using spirometry, we performed sensitivity analyses based on primary care values and UK Biobank values separately.

### Assessing frailty

We used two different definitions of frailty, the frailty index and the frailty phenotype, which we analysed in parallel. These are described briefly here with full details in the online supplemental appendix 1.

A frailty index is a non-weighted count of age-related deficits (including comorbidities, symptoms, functional limitations and laboratory values). The frailty index was originally developed by Rockwood and Mitnitski and includes a standard protocol for selecting deficits from a given data set based on specific criteria.^29–31^ Deficits should be associated with increasing age and with poor health status; be neither too rare (<1% prevalence) or ubiquitous and cover a range of organ systems.^29^ We used the frailty index previously developed by Williams *et al* for UK Biobank.^32^ Deficits are summed and then divided by the total number of possible deficits to give a value between 0 (no deficits) and 1 (all possible deficits). We analysed the frailty index as a numerical variable. For estimating prevalence and for presentation in tables, we also categorised the frailty index into robust (0–0.12), mild (0.12–0.24), moderate (0.24–0.36) and severe (>0.36) frailty. Cut-points were selected based on the electronic frailty index used routinely in UK primary care.^33^

The frailty phenotype is based on five criteria: low grip strength, weight loss, slow walking speed, exhaustion and low physical activity. Frailty is defined as the presence of 3 or more criteria, with 1 or 2 criteria indicating prefrailty. We have previously adapted the original criteria by Fried *et al* to UK Biobank (described in detail elsewhere).^5 7^ Briefly, cut-offs for grip strength were as per the original frailty phenotype description, weight loss was self-reported and (given the wording of the UK Biobank questionnaire) not specified to be ‘unintentional’, slow walking speed was self-reported (in contrast to the original frailty phenotype in which gait speed was measured) as were exhaustion and physical activity. Detailed comparison between the UK Biobank and original definitions for each component are in the online supplemental appendix 1.

### Covariates

Baseline covariates were taken from UK Biobank assessment centre data. Age, sex and ethnicity were self-reported. Body mass index was calculated based on measured height and weight. Smoking was categorised as current, previous and never, based on self-report. Self-reported frequency of alcohol intake was categorised (never/special occasions, 1–3 times per month, 1–4 times per week, of daily/almost daily).

### Outcomes

We assessed the following outcomes by linkage to prospective healthcare records: all-cause mortality; all-cause hospitalisations, MACE; hospitalisation with COPD exacerbation; community COPD exacerbation. Follow-up was 8 years.

Mortality was assessed through linkage to national mortality registers. Hospitalisations were defined as any hospital admission coded as ‘urgent’ or ‘emergency’ (excluding ‘elective’ admissions). MACE was defined using International Classification of Diseases 10th Revision (ICD-10) codes from mortality records (cardiovascular death) and hospital episode statistics (non-fatal myocardial infarction (I21) or stroke (I63-I64)). Hospitalised COPD exacerbations were defined using previously validated ICD-10 codes (acute exacerbation of COPD (J44.0 or J44.1) or lower respiratory tract infection (J22) codes in any position, or COPD code (J44.9) in first position of a hospital episode).^34^

Community COPD exacerbations were identified using a previously validated combination of primary care diagnostic codes, symptom codes and prescriptions.^35^ We defined an exacerbation as either (1) a medical diagnosis of lower respiratory tract infection of acute exacerbation of COPD, (2) prescription of COPD-specific antibiotic combined with oral corticosteroid prescription or (3) two or more respiratory symptoms recorded on the same day as prescription of COPD-specific antibiotics or oral corticosteroids. These criteria were applied after excluding events occurring on the same day as codes suggesting routine annual COPD reviews or provision of rescue medication.^35^

### Statistical analysis

The overall distribution of each frailty measure was summarised descriptively using bar plots. The relationship between frailty and baseline characteristics was summarised using descriptive statistics (means and SD or counts and percentages for continuous and categorical variables, respectively). For the frailty index, we summarised this data using categories of the frailty index (robust, mild, moderate, severe) as described above.

To assess the relationship between each frailty measure and adverse clinical outcomes, we used Cox-proportional hazards models (for all-cause mortality and MACE, modelling time to first event for MACE) and negative binomial models (for all-cause hospitalisations, hospitalised COPD exacerbations and community COPD exacerbations). For MACE, a cause-specific model was used, with participants dying of other causes being censored at death with event status set to ‘0’. All models were initially adjusted for age, sex, socioeconomic status, body mass index, smoking and alcohol frequency (model 1) and then additionally adjusted for FEV1 (expressed as a percentage of predicted FEV1 based on age, height and ethnicity) (model 2). Negative binomial models also included an offset term of log observation time. In all models, fractional polynomials were used to model non-linear associations between numerical variables (frailty index, age, socioeconomic status and percent predicted FEV1). We assessed interactions using product terms between frailty and age and between frailty and percent predicted FEV1. This was to assess whether the association between frailty and outcomes varied depending on age or severity of COPD. Interaction terms were retained if they improved model fit (assessed using Akaike Information Criterion).

In sensitivity analyses, we repeated all of the above analyses restricting the sample to those with primary-care-based spirometry values (as UK Biobank spirometry data were not postbronchodilator). We also repeated all analyses using FEV1 expressed as an absolute value instead of as a percentage of predicted FEV1.

Finally, in post hoc analyses, we modelled the relationship between frailty and mortality, and between frailty and hospital admissions in the full cohort (with available primary care data), including a term for the interaction between frailty and COPD. This was to assess whether any relationship between frailty and mortality or hospitalisation was similar in people with and without COPD.

All analyses were performed using R.

### Patient and public involvement

Patients were not involved in the planning and conduct of this research.

## Results

**Figure 1:**
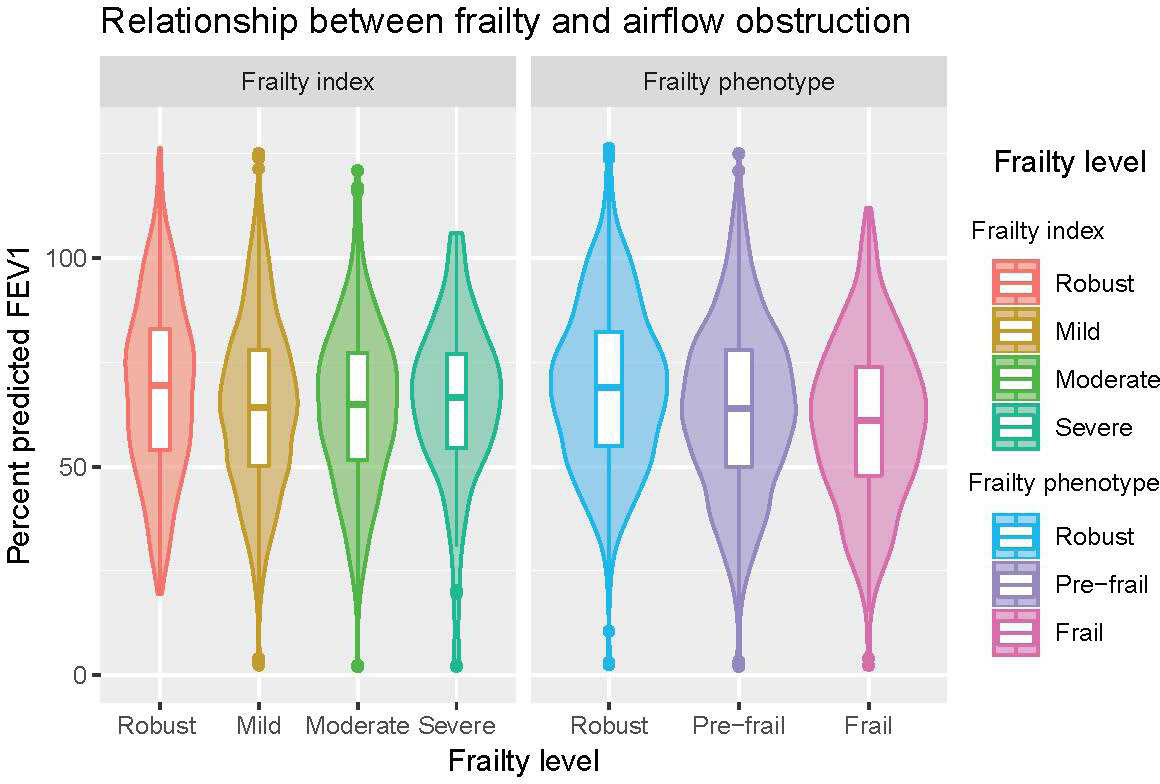
This plot shows the distribution of forced expiratory volume in 1 second (FEV1) values (expressed as a percentage of predicted FEV1 for each individual) stratified by frailty status. The Violin plots show the overall density. The Box plots within these show the median and IQR.

We identified 3132 UK Biobank participants with a COPD-specific primary care diagnostic code prior to baseline assessment (flow diagram shown in online supplemental appendix 1). Of these, 2820 had spirometry data (2203 of which were from primary care data recorded up to 2 years before baseline assessment, with 617 relying on UK Biobank spirometry), 3011 (96%) had complete data on frailty phenotype variables and 3131 (99.9%) had sufficient data to calculate the frailty index. The total number of participants included in each analysis is shown in the flow diagram in online supplemental appendix 1. The prevalence of frailty was 17% (n=514) using the frailty phenotype, while with the frailty index 28% (n=872) had moderate frailty and 4% (n=121) had severe frailty. For both frailty measures, prevalence was higher in people with COPD than in the wider cohort online supplemental appendix 1. Baseline characteristics are shown in table 1. The relationship between frailty and per cent predicted FEV1 is shown in figure 1. Airflow limitation was modestly lower in frailty based on the frailty phenotype (with considerable overlap in the distributions). However, this relationship was not seen between airflow limitation and the frailty index.

**Table 1:** Summary of baseline characteristics of UK Biobank participants with COPD, in total and by frailty status

	Total	Frailty phenotype			Frailty index			
		Robust	Pre-frail	Frail	Robust	Mild frailty	Moderate frailty	Severe frailty
Total N	3131	979	1518	514	467	1671	872	121
Age								
Mean (SD)	61.9 (5.9)	61.7 (5.9)	62.2 (5.8)	61.5 (5.9)	61.4 (6.3)	62.1 (5.9)	61.8 (5.7)	60.8 (5.6)
Sex								
Female (%)	1413 (45.1%)	409 (41.8%)	717 (47.2%)	235 (45.7%)	185 (39.6%)	749 (44.8%)	421 (48.3%)	58 (47.9%)
Male (%)	1718 (54.9%)	570 (58.2%)	801 (52.8%)	279 (54.3%)	282 (60.4%)	922 (55.2%)	451 (51.7%)	63 (52.1%)
Socioeconomic status								
Quintile 1 (most affluent)	367 (11.7%)	174 (17.8%)	158 (10.4%)	25 (4.9%)	79 (16.9%)	218 (13%)	65 (7.5%)	5 (4.1%)
Quintile 2	399 (12.7%)	170 (17.4%)	180 (11.9%)	42 (8.2%)	67 (14.3%)	236 (14.1%)	89 (10.2%)	7 (5.8%)
Quintile 3	520 (16.6%)	202 (20.6%)	240 (15.8%)	60 (11.7%)	97 (20.8%)	285 (17.1%)	121 (13.9%)	17 (14%)
Quintile 4	670 (21.4%)	191 (19.5%)	344 (22.7%)	104 (20.2%)	101 (21.6%)	355 (21.2%)	197 (22.6%)	17 (14%)
Quintile 5 (most deprived)	1170 (37.4%)	241 (24.6%)	593 (39.1%)	282 (54.9%)	121 (25.9%)	575 (34.4%)	399 (45.8%)	75 (62%)
Missing	5	1	3	1	2	2	1	0
Ethnicity								
White	3041 (97.1%)	960 (98.1%)	1478 (97.4%)	499 (97.1%)	446 (95.5%)	1628 (97.4%)	849 (97.4%)	118 (97.5%)
Other	66 (2.9%)	14 (1.9%)	31 (2.6%)	13 (2.9%)	6 (4.5%)	36 (2.6%)	22 (2.6%)	3 (2.5%)
Missing	24	5	9	2	15	7	2	0
BMI								
<18.5	52 (1.7%)	15 (1.5%)	18 (1.2%)	17 (3.3%)	7 (1.5%)	30 (1.8%)	14 (1.6%)	1 (0.8%)
18.5–24.9	853 (27.2%)	307 (31.4%)	410 (27%)	114 (22.2%)	171 (36.6%)	482 (28.8%)	183 (21%)	17 (14%)
25–29.9	1169 (37.3%)	439 (44.8%)	558 (36.8%)	141 (27.4%)	194 (41.5%)	666 (39.9%)	277 (31.8%)	32 (26.4%)
≥30	996 (31.8%)	218 (22.3%)	515 (33.9%)	229 (44.6%)	87 (18.6%)	472 (28.2%)	370 (42.4%)	67 (55.4%)
Missing	61	0	17	13	8	21	28	4
Smoking								
Never	494 (15.8%)	192 (19.6%)	238 (15.7%)	47 (9.1%)	96 (20.6%)	289 (17.3%)	93 (10.7%)	16 (13.2%)
Previous	1628 (52%)	541 (55.3%)	790 (52%)	249 (48.4%)	223 (47.8%)	887 (53.1%)	461 (52.9%)	57 (47.1%)
Current	972 (31%)	238 (24.3%)	477 (31.4%)	211 (41.1%)	134 (28.7%)	482 (28.8%)	309 (35.4%)	47 (38.8%)
	37	8	13	7	14	13	9	1
Alcohol frequency								
Never/special occasions only	914 (29.2%)	194 (19.8%)	436 (28.7%)	237 (46.1%)	86 (18.4%)	427 (25.6%)	347 (39.8%)	54 (44.6%)
One to four times a week	1229 (39.2%)	434 (44.3%)	609 (40.1%)	151 (29.4%)	205 (43.9%)	694 (41.5%)	290 (33.3%)	40 (33.1%)
One to three times a month	327 (10.4%)	108 (11%)	156 (10.3%)	53 (10.3%)	48 (10.3%)	178 (10.7%)	85 (9.7%)	16 (13.2%)
Daily or almost daily	643 (20.5%)	243 (24.8%)	310 (20.4%)	72 (14%)	118 (25.3%)	367 (22%)	147 (16.9%)	11 (9.1%)
Missing	18	0	7	1	10	5	3	0
FEV1 (% predicted)								
>70%	1173 (37.5%)	449 (45.9%)	545 (35.9%)	147 (28.6%)	215 (46%)	607 (36.3%)	309 (35.4%)	42 (34.7%)
50%–70%	1020 (32.6%)	321 (32.8%)	500 (32.9%)	172 (33.5%)	141 (30.2%)	561 (33.6%)	277 (31.8%)	41 (33.9%)
30%–50%	518 (16.5%)	136 (13.9%)	264 (17.4%)	100 (19.5%)	68 (14.6%)	300 (18%)	141 (16.2%)	9 (7.4%)
<30%	109 (3.5%)	11 (1.1%)	65 (4.3%)	26 (5.1%)	15 (3.2%)	60 (3.6%)	30 (3.4%)	4 (3.3%)
Missing	311	62	144	69	28	143	115	25
Medication								
Inhaled corticosteroid	1888 (60.3%)	547 (55.9%)	941 (62.0%)	334 (65.0%)	233 (49.9%)	1027 (61.5%)	556 (63.8%)	72 (59.5%)
LABA	2315 (73.9%)	668 (68.2%)	1158 (76.3%)	403 (78.4%)	289 (61.9%)	1241 (74.3%)	686 (78.7%)	99 (81.8%)
LAMA	1896 (60.6%)	481 (49.1%)	968 (63.8%)	375 (73.0%)	213 (45.6%)	1006 (60.2%)	582 (66.7%)	95 (78.5%)

*Based on prescription within the 6 months prior to baseline.

BMI, body mass index; LABA, long-acting beta-agonist; LAMA, long-acting muscarinic antagonist.

**Figure 2:**
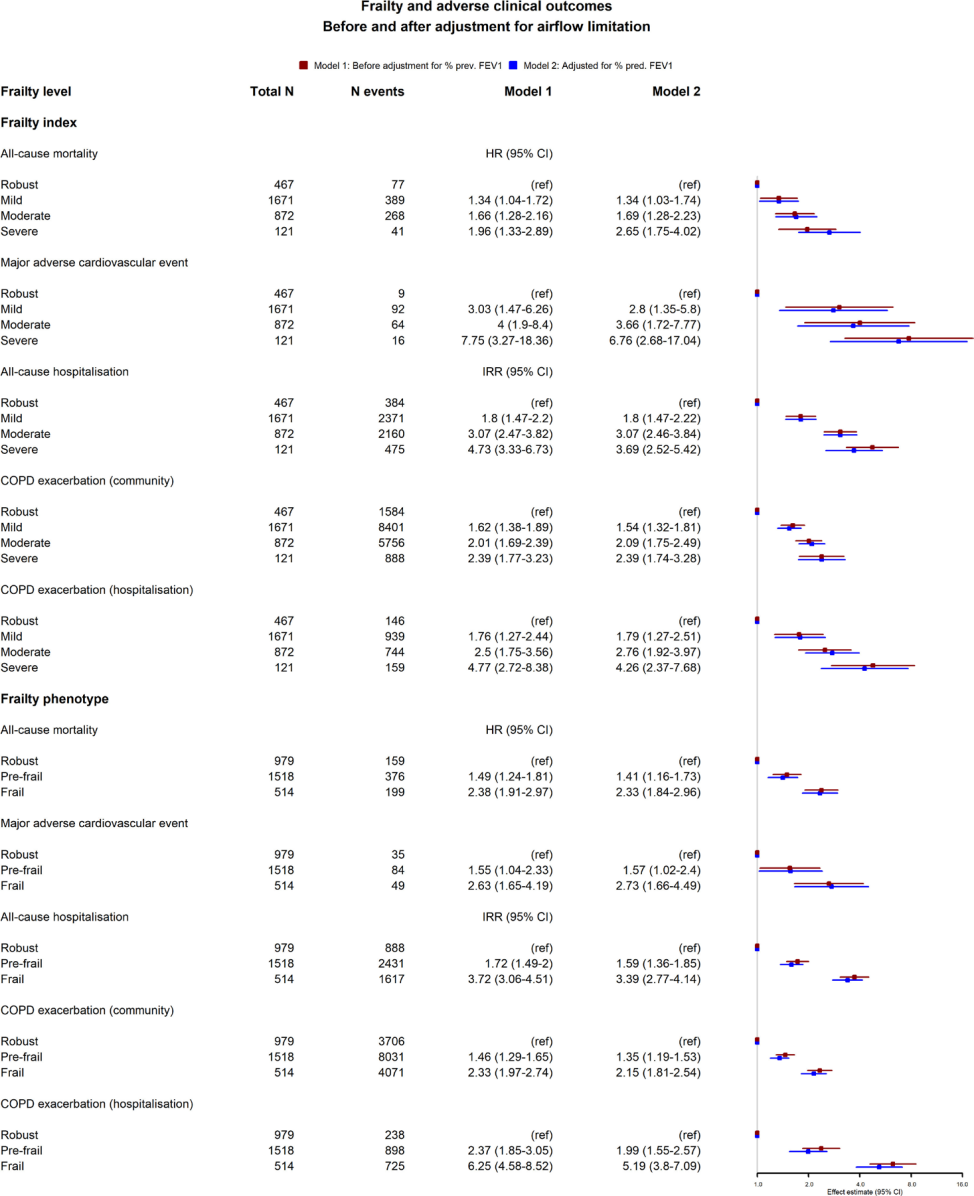
This figure shows HR and incidence rate ratios (IRR) for the association between frailty and clinical outcomes. Two models are presented, model 1 (adjusted for age, sex, socioeconomic status, smoking and alcohol frequency) and model 2 (adjusted for all covariates in model one plus forced expiratory volume in 1 s).

The relationship between frailty and clinical outcomes is summarised in figure 2. Using both the frailty index and the frailty phenotype definition, presence of frailty was associated with greater risk of all-cause mortality, MACE, all-cause hospitalisations, hospitalisation with COPD exacerbation and community COPD exacerbation. For MACE, CIs for different levels of frailty index, and for prefrailty and frailty, were overlapping. The relative effect of frailty on each of these outcomes was similar before and after adjusting for airflow limitation, with only modest attenuation of the effect estimates.

**Figure 3:**
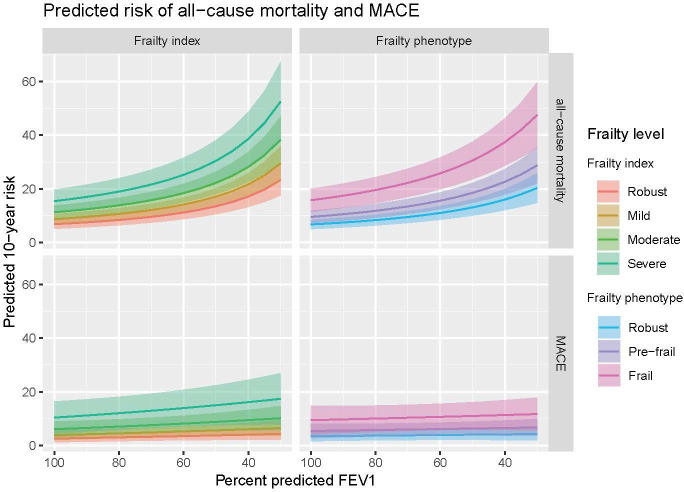
This plot shows the predicted 10-year risk of all-cause mortality (top two panels) and major adverse cardiovascular events (bottom two panels) based on frailty status and forced expiratory volume in 1 second (FEV1). Coloured lines indicate the point estimates for each level of frailty, with shaded areas showing the corresponding 95% CIs. Results adjusted for age, sex, socioeconomic status, smoking and alcohol frequency. MACE, major adverse cardiovascular event.

**Figure 4:**
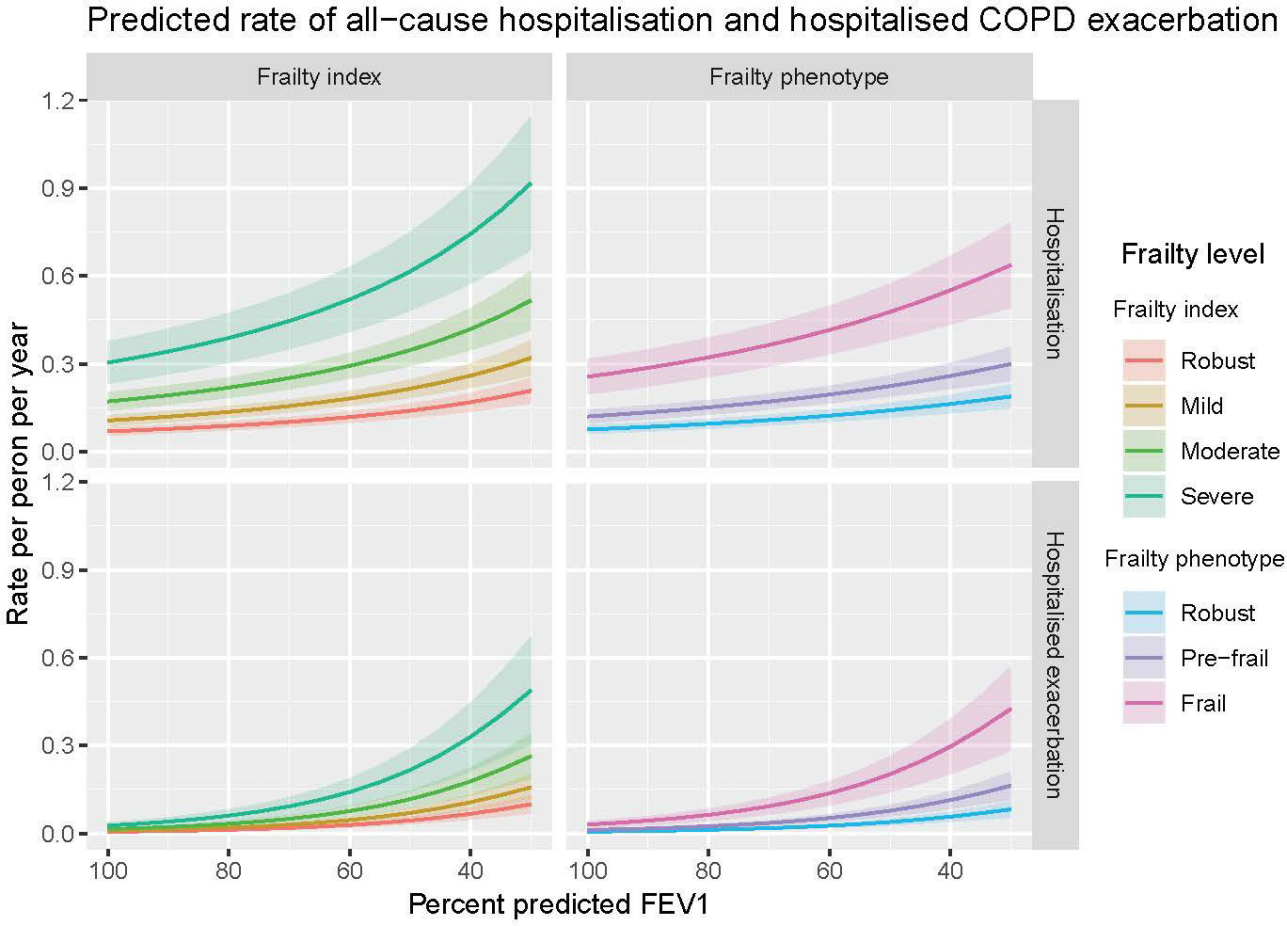
This plot shows the predicted 10-year risk of all-cause hospitalisation (top two panels) and hospitalisation due to COPD exacerbation (bottom two panels) based on frailty status and forced expiratory volume in 1 second (FEV1). Coloured lines indicate the point estimates for each level of frailty, with shaded areas showing the corresponding 95% CIs. Results adjusted for age, sex, socioeconomic status, smoking and alcohol frequency. COPD, chronic obstructive pulmonary disease.

The predicted risk of clinical outcomes at different levels of frailty and airflow obstruction are shown in figure 3 (all-cause mortality and MACE), figure 4 (all-cause hospitalisation and hospitalised COPD exacerbations) and online supplemental appendix 1 (community COPD exacerbations).

At all levels of frailty, the risk of all-cause mortality rose in a non-linear fashion with lower FEV1. There was no evidence of statistical interaction between either frailty definition and FEV1 or between age and either frailty or FEV1. This implies that, although the relative increase in mortality risk with frailty was similar at all levels of airflow obstruction, the absolute difference in mortality risk between ‘robust’ and ‘frail’ individuals was greatest in participants with lower FEV1. Furthermore, although the relative impact of frailty did not vary with age, absolute risk of outcomes is also therefore greater among older participants at any given level of frailty.

For MACE, the relationship with airflow limitation, as well as with frailty, was more modest. However, both were independently associated with a higher risk of MACE.

For hospitalisations and COPD exacerbations (hospitalised or community), there was a clear increase in risk with both airflow limitation and with frailty (figure 4 and online supplemental appendix 1). As with mortality and MACE, there was no evidence of statistical interaction.

In sensitivity analyses based on primary care-coded spirometry data, all results were similar including the relationship between frailty and FEV1 and the relationship between frailty and clinical outcomes adjusting for FEV1. Findings were also similar when using raw FEV1 values rather than per cent-predicted FEV1. Finally, the relationship between frailty and mortality and between frailty and hospital admissions, on the relative scale, was similar between people with and without COPD (with no evidence of statistical interaction, shown in the online supplemental appendix 1).

## Discussion

Frailty is common in ‘middle-aged’ as well as older people with COPD and is associated with a range of adverse health outcomes. In UK Biobank participants with COPD, aged between 40 and 70, frailty prevalence was 17% using the frailty phenotype, while using the frailty index 28% had moderate and 4% had severe frailty. The frailty phenotype, but not the frailty index, was associated with lower percent-predicted FEV1. Both frailty definitions were associated with higher all-cause mortality, MACE, hospitalisations and both hospitalised and community COPD exacerbations. The relationship with each of these adverse outcomes was independent of the degree of airflow limitation, for both frailty definitions. However, the difference in absolute risk between frail and robust participants was greatest in those with severe airflow limitation. These findings demonstrate that frailty is a common and clinically significant concept in people with COPD, including those aged <65 years in whom it is not routinely identified and has been infrequently studied.

Our findings that frailty in COPD is associated with mortality independently of FEV1 are consistent with some previous studies,^19 21^ although some have shown null associations after adjustment for age and FEV1.^22 23^ These studies varied in their frailty definition, sample size and length of follow-up. Frailty has also been associated with exacerbations in two cross-sectional and one longitudinal study.^18 21 36^ The association with MACE has not been described in previous studies of frailty in COPD.

Our findings that frailty was common in people with COPD are in keeping with previous epidemiological studies of frailty in COPD^12^ as well as the wider literature of the broad physiological implications of COPD.^37^ COPD impacts multiple organ systems and is often associated with muscle weakness, osteoporosis and malnutrition.^10 11^ The severity of COPD is best characterised by a multidimentional assessment reflecting these broad impacts. For example, the BODE (Body-mass index, airflow Obstruction, Dyspnea, and Exercise) index comprises four domains (body mass index, FEV1, dyspnoea assessed using the modified Medical Research Council scale and exercise capacity based on the 6 min walking distance). It is used to assess the severity of COPD, and it is a superior predictor of mortality in COPD than FEV1 alone.^38^ Domains of the BODE index have considerable overlap with features of the frailty phenotype (eg, weight loss and slow walking speed) and are commonly-used deficits within the frailty index. However, the extent to which frailty is caused by these features of COPD, or reflects a physiological decline distinct from COPD, is not clear. The development of frailty is multifactorial with multiple potential causal mechanisms. Many of these, including environmental exposures, systemic inflammation and altered body composition, are closely linked to COPD (either as common causal factors, such as environmental exposures, or as sequelae of COPD that may contribute to the development of frailty). As frailty development is multifactorial, this is likely to vary between individuals and may also differ depending on the measure used to define frailty.

Frailty is a dynamic concept. Longitudinal studies have shown that COPD is associated with the transition from a robust to a frail state using the frailty phenotype.^39 40^ Conversely, some people with frailty and COPD undergoing pulmonary rehabilitation show a marked improvement in frailty status.^41^ Therefore, while COPD may be a risk factor for frailty progression, the shared features may offer opportunities for interventions targeting both frailty status and COPD. The observation that frailty may improve in the context of pulmonary rehabilitation, as described by Maddocks *et al*,^41^ is consistent with recent reviews of interventions targeting frailty in general, in which exercise and nutritional interventions have shown the most promise in ameliorating frailty.^42^ Identification of people with COPD and frailty may, therefore, be beneficial for both identification of risk and for targeted intervention. Our findings demonstrate that this identification should not be limited to ‘older’ people with COPD, as frailty is prevalent across a wide age range and associated with a range of clinically important outcomes.

Strengths of this study include its large sample size and prospective linkage to a wide range of healthcare outcomes. We also used validated definitions, based on linked diagnostic codes, to identify baseline COPD and subsequent exacerbations.^26 34 35^ The range of variables available from the UK Biobank baseline assessment also allows the analysis of two separate measures of frailty. However, there are some important limitations. Our definition of the frailty phenotype was adapted from the original.^5 7^ Unlike the original, weight loss was not specified as unintentional in UK Biobank and walking speed was self-reported rather than measured. The frailty index was constructed according to the standard protocol; however, there is a relative lack of functional measures and few measures of sensory or cognitive impairment. UK Biobank is also not nationally representative, with participants being on average more affluent, having fewer comorbidities, and more predominantly White ethnicity than the UK population. This lack of representativeness may lead to bias in the estimation of associations between exposure and outcomes. For example, UK Biobank appears to underestimate the risks of mortality, hospitalisation and MACEs associated with high levels of multimorbidity.^43^ It is likely, therefore, that our estimates of the associations between frailty and adverse outcomes may be conservative. UK Biobank spirometry data are also not postbronchodilator; however, we used primary care spirometry data where possible (available for 70% of participants), which has been shown to be of high quality, and our findings were consistent when restricting our analysis to those with primary care spirometry alone.

### Conclusion

Our findings demonstrate that frailty is common in people with COPD, including those under 65 years of age, and has clinically significant implications for this population regardless of which frailty definition is used. This relationship is independent of the degree of airflow limitation. Identification of frailty in people with COPD may aid risk stratification and identification of those who may benefit from targeted interventions. For this to be beneficial, frailty assessment would need to become integrated into the routine monitoring and management of COPD.

## Data Availability

Data may be obtained from a third party and are not publicly available. The UK Biobank data that support the findings of this study are available from the UK Biobank (www.ukbiobank.ac.uk), subject to approval by UK Biobank.
