# Supplementary material for: Frailty in COPD: an analysis of prevalence and clinical impact using UK Biobank
Source: BMJ Open Respir Res. 2022 Jul 4;9(1):e001314. doi: 10.1136/bmjresp-2022-001314 (PMC9255399; doi:10.1136/bmjresp-2022-001314)
Supplement: Supplementary data [file bmjresp-2022-001314supp001.pdf]

Supplementary appendix: Frailty in COPD: an analysis of prevalence and clinical impact using UK Biobank

Peter Hanlon, Jim Lewsey, Jennifer Quint, Bhautesh D Jani, Barbara I Nicholl, David A McAllister, Frances S Mair.

Contents

UK Biobank: Comparison of participants with linked GP data versus those without GP data available 2
Quantification of frailty index 2
Quantification of the frailty phenotype 4
Selection of participants 5
Comparison of frailty prevalence with full cohort 6
Prevalence of domains of frailty phenotype 6
Distributions of frailty 7
Scatter plot of frailty index (numerical values) and percent predicted FEV1 7
Comparing distribution of airflow limitation using primary care spirometry and UK Biobank spirometry 8
Relationship between frailty and clinical outcomes, adjusting for primary care spirometry only 9
Relationship between frailty and clinical outcomes, adjusting for UK Biobank spirometry only 10
Relationship between frailty, FEV1 and outcomes, comparing main analysis to UK Biobank spirometry only 11

UK Biobank: Comparison of participants with linked GP data versus those without GP data available

	Whole cohort (n=502,533)	GP data available (n=211,597)	No GP data available (n=290,936)
Mean age (sd)	56.5 (8.1)	56.5 (8.1)	56.5 (8.1)
Sex (%)			
Male	229,132 (45.6%)	96,060 (45.4%)	133,072 (45.7%)
Female	273,401 (54.4%)	115,537 (54.6%)	157,864 (54.3%)
Socioeconomic status			
Quintile 1 (most affluent)	100,663 (20.1%)	42,155 (20.0%)	58,508 (20.1%)
2	100,096 (19.9%)	41,628 (19.7%)	58,468 (20.1%)
3	100,398 (20.0%)	43,378 (20.5%)	57,020 (19.6%)
4	100,375 (20.0%)	42,531 (20.1%)	57,844 (19.9%)
Quintile 5 (most deprived)	100,378 (20.0%)	41,581 (19.7%)	58,797 (20.2%)
Self-reported LTC count*			
0	172,565 (34.5%)	71,572 (34.0%)	100,993 (34.8%)
1	163,680 (32.7%)	68,987 (32.7%)	94,693 (32.7%)
2	95,211 (19.0%)	40,353 (19.1%)	54,858 (18.9%)
3	43,113 (8.6%)	18,702 (8.9%)	24,411 (8.4%)
4	16,732 (3.3%)	7,175 (3.4%)	9,557 (3.3%)
5	6,056 (1.2%)	2,580 (1.2%)	3,476 (1.2%)
6 or more	3,331 (0.7%)	1,428 (0.7%)	1,903 (0.7%)
Note that the LTC count displayed here is based on baseline assessment centre self-report of LTCs, with conditions based on the original list of conditions used in the main analysis, adapted for UK Biobank baseline self-reported data. These definitions were not used in the main analysis as equivalent (self-reported) data are not available for SAIL.			

Quantification of frailty index

Frailty index deficits taken from Williams DM, Jylhävä J, Pedersen NL, Hägg S. A frailty index for UK Biobank participants. The Journals of Gerontology: Series A. 2019 Mar 14;74(4):582-7.

https://doi.org/10.1093/gerona/gly094

**Deficit**	**Coding**
Glaucoma *	Categorised 0/1
Cataracts *	Categorised 0/1
Hearing difficulty	Categorised 0/1
Migraine *	Categorised 0/1
Dental problems	Categorised 0/1 for none vs. any
Self-rated health	0 – excellent; 0.25 – good; 0.5 - fair, 1 - poor
Fatigue: frequency of tiredness / lethargy in last two weeks	0, 0.25, 0.5, 1, respectively
Sleep: experience of sleeplessness/insomnia	Categorised 0, 0.5, 1, respectively

Depressed feelings: frequency in last two weeks	0 – not at all, 0.5 – several days, 0.75 -- more than half, 1 – nearly every day
Self-described nervous personality	Categorised 0/1
Severe anxiety/ panic attacks *	Categorised 0/1
Common to feel loneliness	Categorised 0/1
Sense of misery (ever/never)	Categorised 0/1
Infirmity: long-standing illness or disability	Categorised 0/1
Falls in last year	0 - no fall, 0.5 - one fall, 1 - more than one fall
Fractures/broken bones in last five years	Categorised 0/1
Diabetes *	Categorised 0/1
Myocardial infarction *	Categorised 0/1
Angina *	Categorised 0/1
Stroke *	Categorised 0/1
High blood pressure *	Categorised 0/1
Hypothyroidism *	Categorised 0/1
Deep-vein thrombosis *	Categorised 0/1
High cholesterol *	Categorised 0/1
Breathing: wheeze in last year	Categorised 0/1
Pneumonia *	Categorised 0/1
Chronic bronchitis/emphysema *	Categorised 0/1
Asthma *	Categorised 0/1
Rheumatoid arthritis *	Categorised 0/1
Osteoarthritis *	Categorised 0/1
Gout *	Categorised 0/1
Osteoporosis *	Categorised 0/1
Hayfever, allergic rhinitis or eczema *	Categorised 0/1
Psoriasis *	Categorised 0/1
Any cancer diagnosis *	Categorised 0/1
Multiple cancers diagnosed (number reported)	Categorised 0/1
Chest pain	Categorised 0/1
Head and/or neck pain	Categorised 0/1
Back pain	Categorised 0/1
Stomach/abdominal pain	Categorised 0/1
Hip pain	Categorised 0/1
Knee pain	Categorised 0/1
Whole-body pain	Categorised 0/1
Facial pain	Categorised 0/1
Sciatica *	Categorised 0/1
Gastric reflux *	Categorised 0/1
Hiatus hernia *	Categorised 0/1
Gall stones *	Categorised 0/1
Diverticulitis *	Categorised 0/1

Quantification of the frailty phenotype

Taken from Hanlon P, Nicholl BI, Jani BD, Lee D, McQueenie R, Mair FS. Frailty and pre-frailty in middle-aged and older adults and its association with multimorbidity and mortality: a prospective analysis of 493 737 UK Biobank participants. The Lancet Public Health. 2018 Jul 1;3(7):e323-32.

https://doi.org/10.1016/S2468-2667(18)30091-4

Frailty phenotype variable definitions adapted for UK Biobank	
Weight loss	Self-reported: "Compared with one year ago, has your weight changed?" (response: yes, lost weight=1, other=0)
Exhaustion	Self-reported: "Over the past two weeks, how often have you felt tired or had little energy?"<br>(response: more than half the days or nearly every day=1, other=0)
Low physical activity	Self-reported: UK Biobank physical activity questionnaire. We classified the responses into: none (no physical activity in the last 4 weeks), low (light DIY activity [eg, pruning, watering the lawn] only in the past 4 weeks), medium (heavy DIY activity [eg, weeding, lawn mowing, carpentry and digging], walking for pleasure, or other exercises in the past 4 weeks), and high (strenuous sports in the past 4 weeks)<br>(response: none or light activity with a frequency of once per week or less=1, medium or heavy activity, or light activity more than once per week=0)
Slow walking pace	Self-reported: "How would you describe your usual walking pace?" (response: slow=1, other=0)
Low grip strength	Measured grip strength (sex and body-mass index adjusted cutoffs taken from Fried et al)

Selection of participants

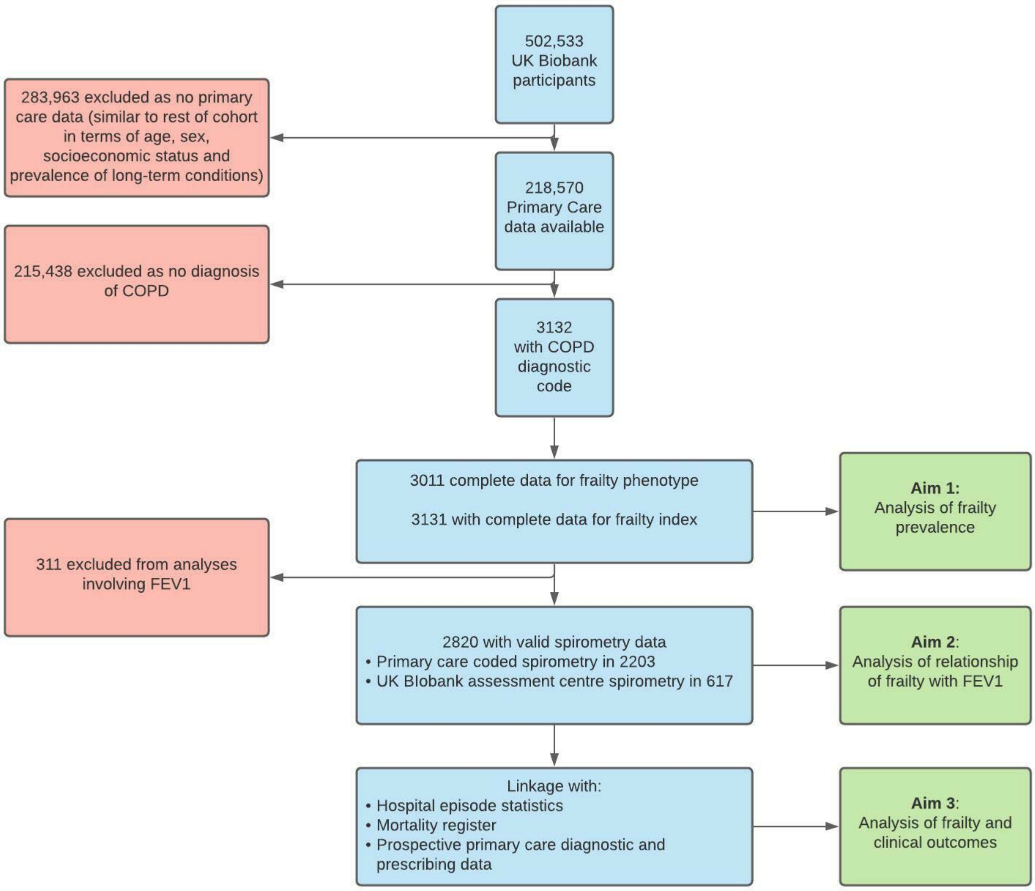

Comparison of frailty prevalence with full cohort

	COPD	No COPD
Total	3132	215439
Frailty phenotype		
Robust	979 (31.3%)	125207 (58.1%)
Pre-frail	1518 (48.5%)	79484 (36.9%)
Frail	514 (16.4%)	7319 (3.4%)
Missing	121	3429
Frailty index		
Robust	467 (14.9%)	112289 (52.1%)
Mild	1671 (53.4%)	88142 (40.9%)
Moderate	872 (27.9%)	13919 (6.5%)
Severe	121 (3.9%)	1089 (0.5%)
Missing	1	349

Prevalence of domains of frailty phenotype

Domain	Total with deficit (%)	Total missing
Low grip strength	870 (28.3%)	55
Weight loss	547 (17.5%)	9
Exhaustion	748 (23.9%)	8
Slow walking speed	1098 (35.8%)	62
Low physical activity	709 (23.2%)	71

Distributions of frailty

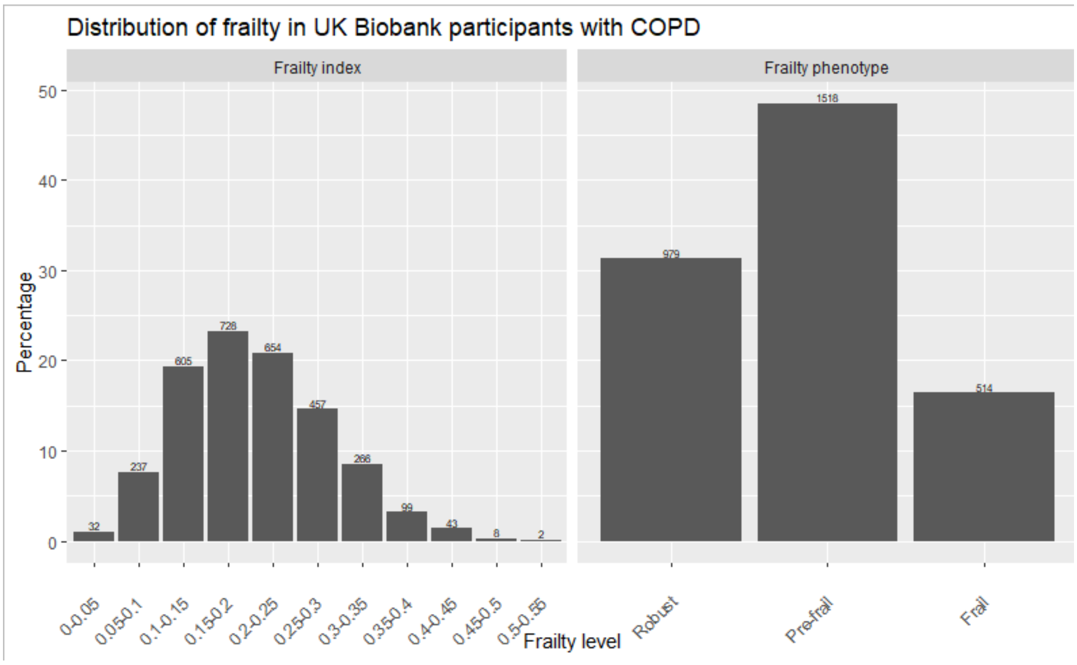

Legend: Bar plot showing the distribution of each frailty measure in UK Biobank participants with COPD. The numbers above the bars indicate the total number of participants with each frailty status/frailty index value.

Scatter plot of frailty index (numerical values) and percent predicted FEV1

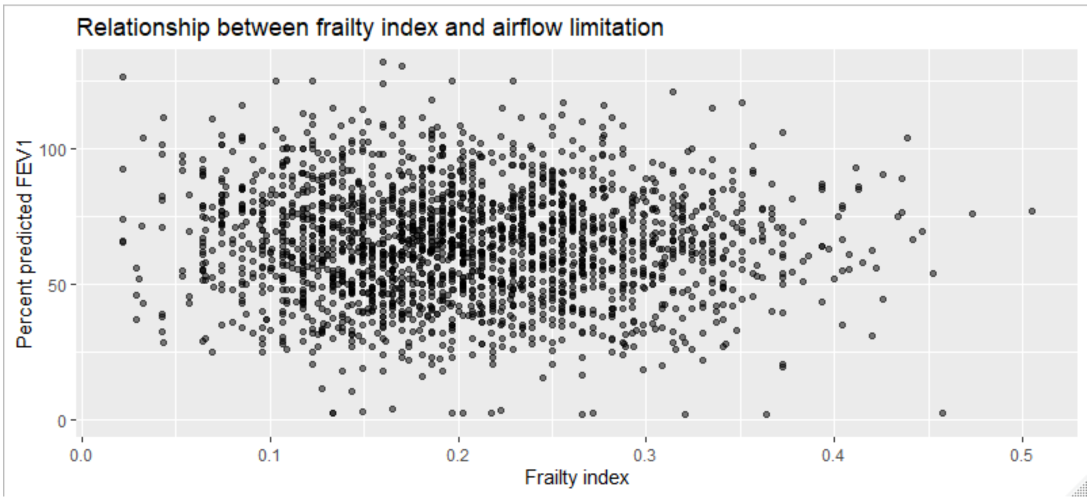

Comparing distribution of airflow limitation using primary care spirometry and UK Biobank spirometry

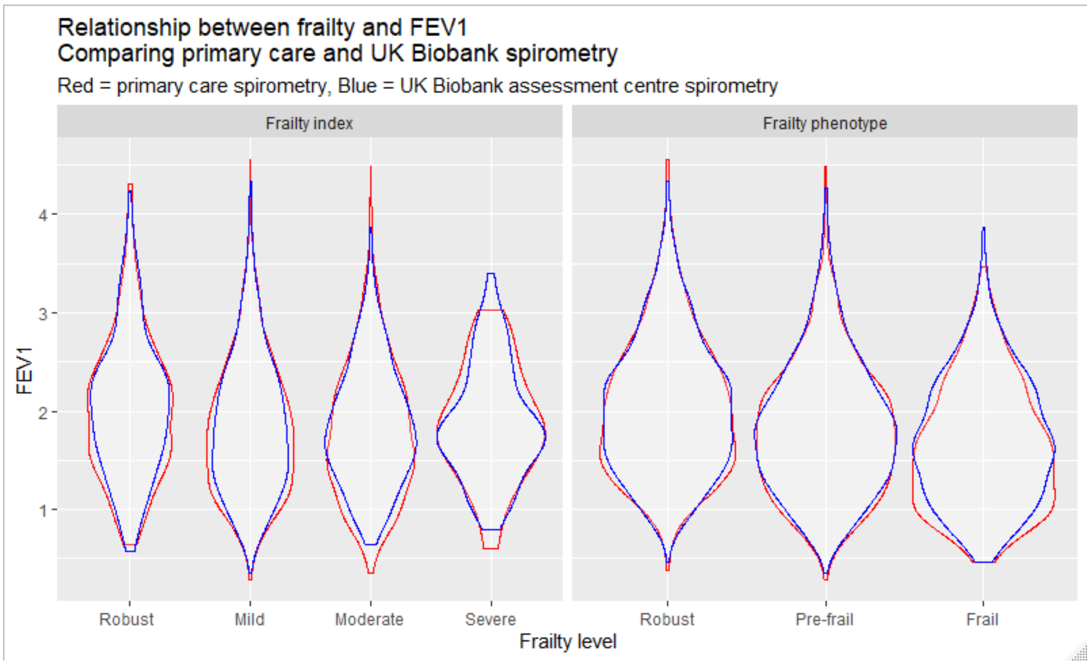

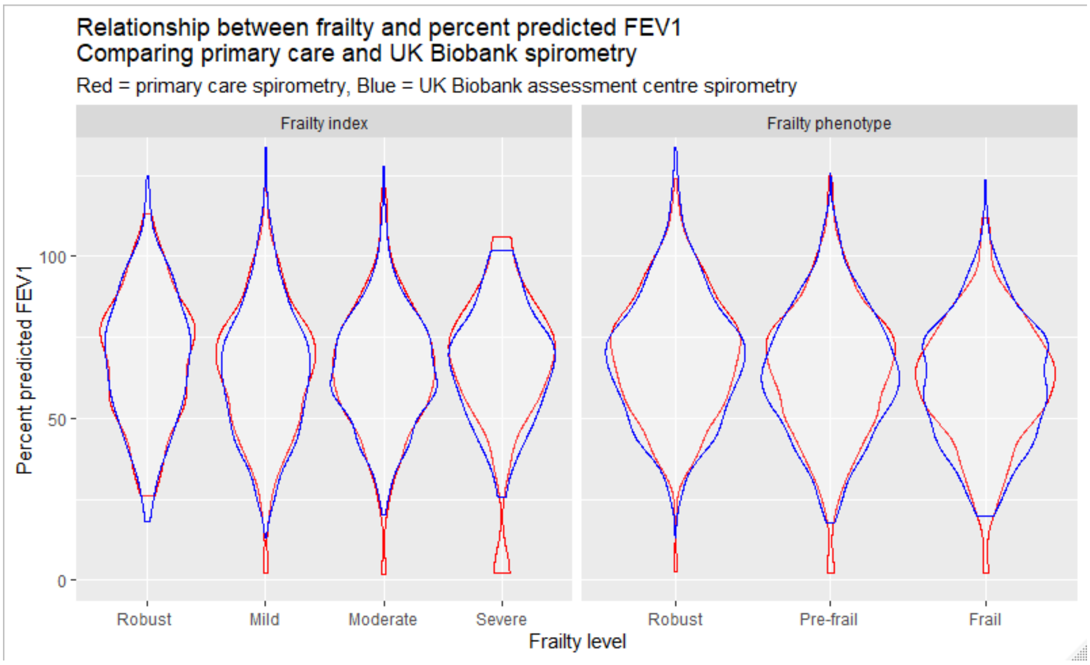

Relationship between frailty and clinical outcomes, adjusting for primary care spirometry only

Frailty and adverse clinical outcomes
Before and after adjustment for airflow limitation (primary care data only)

■ Model 1: Before adjustment for % prev. FEV1 ■ Model 2: Adjusted for % pred. FEV1

Frailty level	Total N	N events	Model 1	Model 2
**Frailty index**				
All−cause mortality			HR (95% CI)	
Robust	467	77	(ref)	(ref)
Mild	1671	389	1.34 (1.04−1.72)	1.39 (1.04−1.87)
Moderate	872	268	1.66 (1.28−2.16)	1.75 (1.28−2.39)
Severe	121	41	1.96 (1.33−2.89)	2.54 (1.57−4.1)
Major adverse cardiovascular event				
Robust	467	9	(ref)	(ref)
Mild	1671	92	3.03 (1.47−6.26)	3.42 (1.37−8.53)
Moderate	872	64	4 (1.9−8.4)	4.33 (1.7−11.07)
Severe	121	16	7.75 (3.27−18.36)	7.93 (2.55−24.68)
All−cause hospitalisation			IRR (95% CI)	
Robust	467	384	(ref)	(ref)
Mild	1671	2371	1.8 (1.47−2.2)	1.68 (1.34−2.11)
Moderate	872	2160	3.07 (2.47−3.82)	2.86 (2.24−3.66)
Severe	121	475	4.73 (3.33−6.73)	3.22 (2.09−4.97)
COPD exacerbation (community)				
Robust	467	1584	(ref)	(ref)
Mild	1671	8401	1.62 (1.38−1.89)	1.46 (1.22−1.74)
Moderate	872	5756	2.01 (1.69−2.39)	2.09 (1.72−2.55)
Severe	121	888	2.39 (1.77−3.23)	2.3 (1.59−3.31)
COPD exacerbation (hospitalisation)				
Robust	467	146	(ref)	(ref)
Mild	1671	939	1.76 (1.27−2.44)	1.79 (1.23−2.6)
Moderate	872	744	2.5 (1.75−3.56)	2.83 (1.89−4.22)
Severe	121	159	4.77 (2.72−8.38)	3.68 (1.88−7.2)
**Frailty phenotype**				
All−cause mortality			HR (95% CI)	
Robust	979	159	(ref)	(ref)
Pre−frail	1518	376	1.49 (1.24−1.81)	1.37 (1.1−1.71)
Frail	514	199	2.38 (1.91−2.97)	2.26 (1.74−2.93)
Major adverse cardiovascular event				
Robust	979	35	(ref)	(ref)
Pre−frail	1518	84	1.55 (1.04−2.33)	1.27 (0.8−2.03)
Frail	514	49	2.63 (1.65−4.19)	2.15 (1.24−3.73)
All−cause hospitalisation			IRR (95% CI)	
Robust	979	888	(ref)	(ref)
Pre−frail	1518	2431	1.72 (1.49−2)	1.55 (1.31−1.83)
Frail	514	1617	3.72 (3.06−4.51)	3.29 (2.64−4.1)
COPD exacerbation (community)				
Robust	979	3706	(ref)	(ref)
Pre−frail	1518	8031	1.46 (1.29−1.65)	1.38 (1.2−1.58)
Frail	514	4071	2.33 (1.97−2.74)	2.32 (1.92−2.79)
COPD exacerbation (hospitalisation)				
Robust	979	238	(ref)	(ref)
Pre−frail	1518	898	2.37 (1.85−3.05)	2.02 (1.54−2.66)
Frail	514	725	6.25 (4.58−8.52)	5.19 (3.7−7.28)

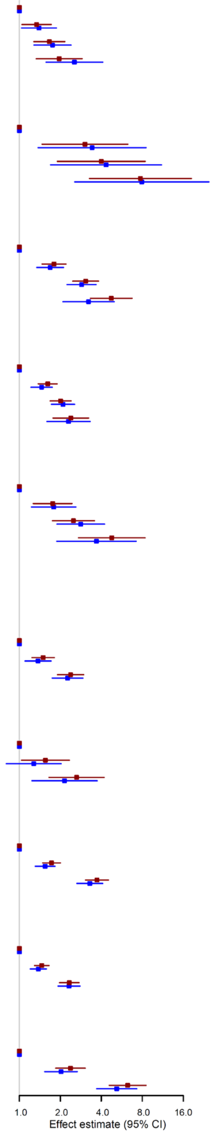

Relationship between frailty and clinical outcomes, adjusting for UK Biobank spirometry only

Frailty and adverse clinical outcomes
Before and after adjustment for airflow limitation (UK Biobank spirometry data only)

Model 1: Before adjustment for % prev. FEV1 | Model 2: Adjusted for % pred. FEV1

Frailty level	Total N	N events	Model 1	Model 2
**Frailty index**				
All−cause mortality			HR (95% CI)	
Robust	467	77	(ref)	(ref)
Mild	1671	389	1.34 (1.04−1.72)	1.23 (0.91−1.66)
Moderate	872	268	1.66 (1.28−2.16)	1.63 (1.18−2.25)
Severe	121	41	1.96 (1.33−2.89)	2.23 (1.34−3.69)
Major adverse cardiovascular event				
Robust	467	9	(ref)	(ref)
Mild	1671	92	3.03 (1.47−6.26)	2.45 (1.11−5.4)
Moderate	872	64	4 (1.9−8.4)	3.21 (1.4−7.33)
Severe	121	16	7.75 (3.27−18.36)	5.88 (2.03−17.01)
All−cause hospitalisation			IRR (95% CI)	
Robust	467	384	(ref)	(ref)
Mild	1671	2371	1.8 (1.47−2.2)	1.69 (1.34−2.14)
Moderate	872	2160	3.07 (2.47−3.82)	3.16 (2.44−4.11)
Severe	121	475	4.73 (3.33−6.73)	3.36 (2.1−5.37)
COPD exacerbation (community)				
Robust	467	1584	(ref)	(ref)
Mild	1671	8401	1.62 (1.38−1.89)	1.47 (1.23−1.76)
Moderate	872	5756	2.01 (1.69−2.39)	2.08 (1.69−2.55)
Severe	121	888	2.39 (1.77−3.23)	2.14 (1.46−3.14)
COPD exacerbation (hospitalisation)				
Robust	467	146	(ref)	(ref)
Mild	1671	939	1.76 (1.27−2.44)	1.46 (0.98−2.16)
Moderate	872	744	2.5 (1.75−3.56)	3.01 (1.96−4.61)
Severe	121	159	4.77 (2.72−8.38)	3.64 (1.75−7.56)
**Frailty phenotype**				
All−cause mortality			HR (95% CI)	
Robust	979	159	(ref)	(ref)
Pre−frail	1518	376	1.49 (1.24−1.81)	1.24 (0.99−1.56)
Frail	514	199	2.38 (1.91−2.97)	2.01 (1.52−2.66)
Major adverse cardiovascular event				
Robust	979	35	(ref)	(ref)
Pre−frail	1518	84	1.55 (1.04−2.33)	1.66 (1−2.77)
Frail	514	49	2.63 (1.65−4.19)	3.35 (1.83−6.1)
All−cause hospitalisation			IRR (95% CI)	
Robust	979	888	(ref)	(ref)
Pre−frail	1518	2431	1.72 (1.49−2)	1.64 (1.37−1.96)
Frail	514	1617	3.72 (3.06−4.51)	3.38 (2.63−4.35)
COPD exacerbation (community)				
Robust	979	3706	(ref)	(ref)
Pre−frail	1518	8031	1.46 (1.29−1.65)	1.21 (1.05−1.4)
Frail	514	4071	2.33 (1.97−2.74)	1.89 (1.53−2.33)
COPD exacerbation (hospitalisation)				
Robust	979	238	(ref)	(ref)
Pre−frail	1518	898	2.37 (1.85−3.05)	1.83 (1.35−2.48)
Frail	514	725	6.25 (4.58−8.52)	4.85 (3.27−7.19)

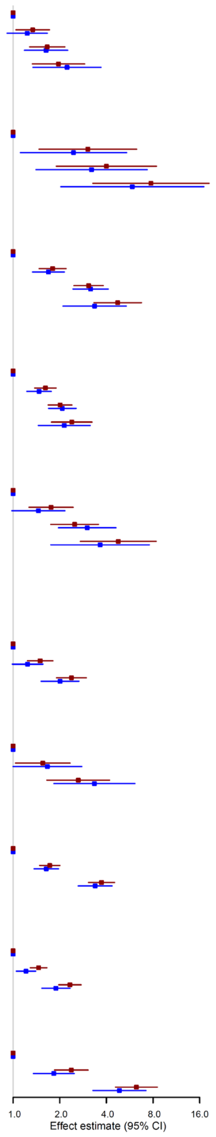

Relationship between frailty, FEV1 and outcomes, comparing main analysis to UK Biobank spirometry only

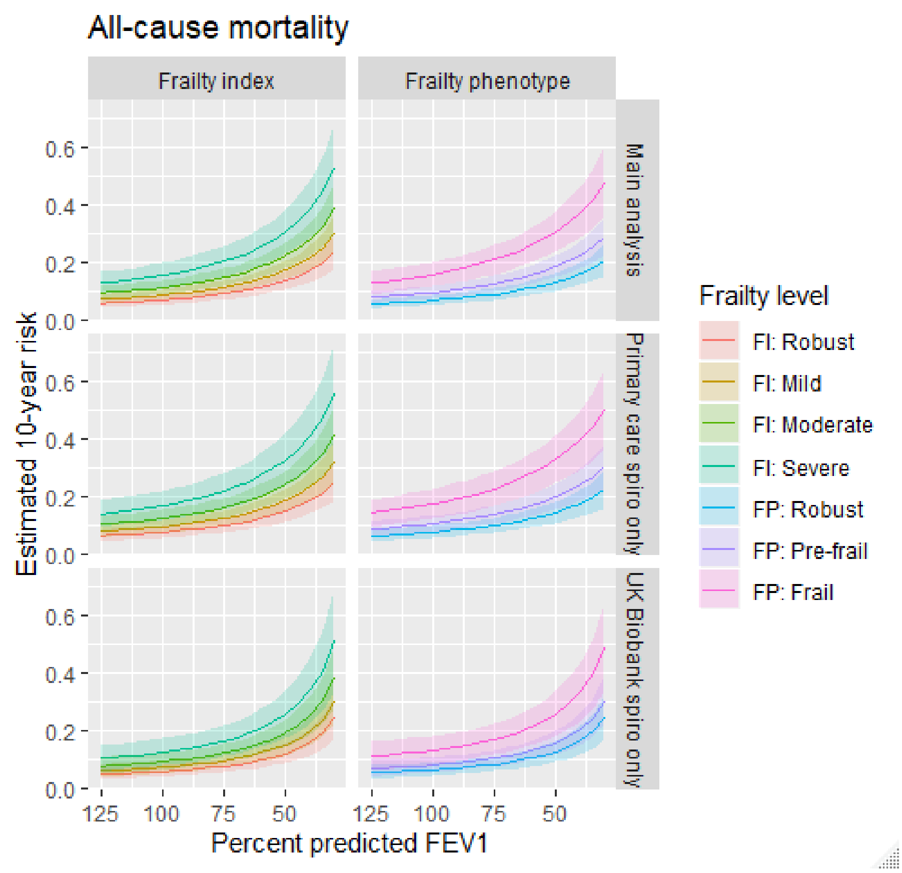

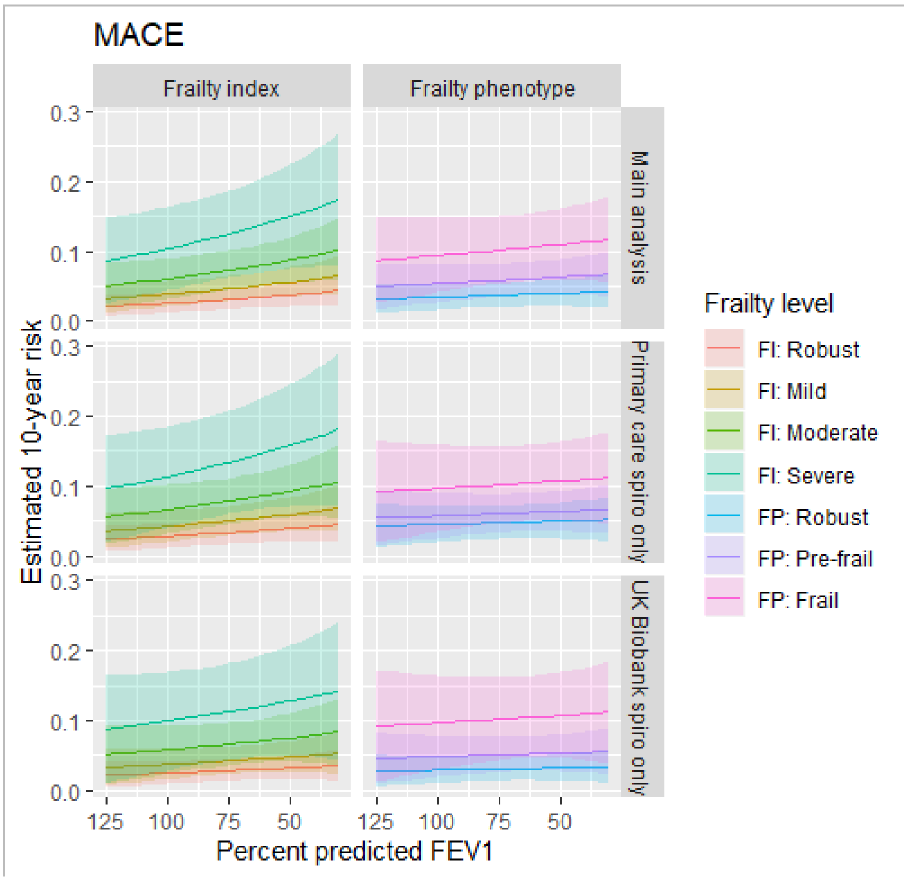
MACE
Frailty index
Frailty phenotype
Main analysis
Primary care spiro only
UK Biobank spiro only
Estimated 10-year risk
0.0
0.1
0.2
0.3
125
100
75
50
Percent predicted FEV1
Frailty level
FI: Robust
FI: Mild
FI: Moderate
FI: Severe
FP: Robust
FP: Pre-frail
FP: Frail

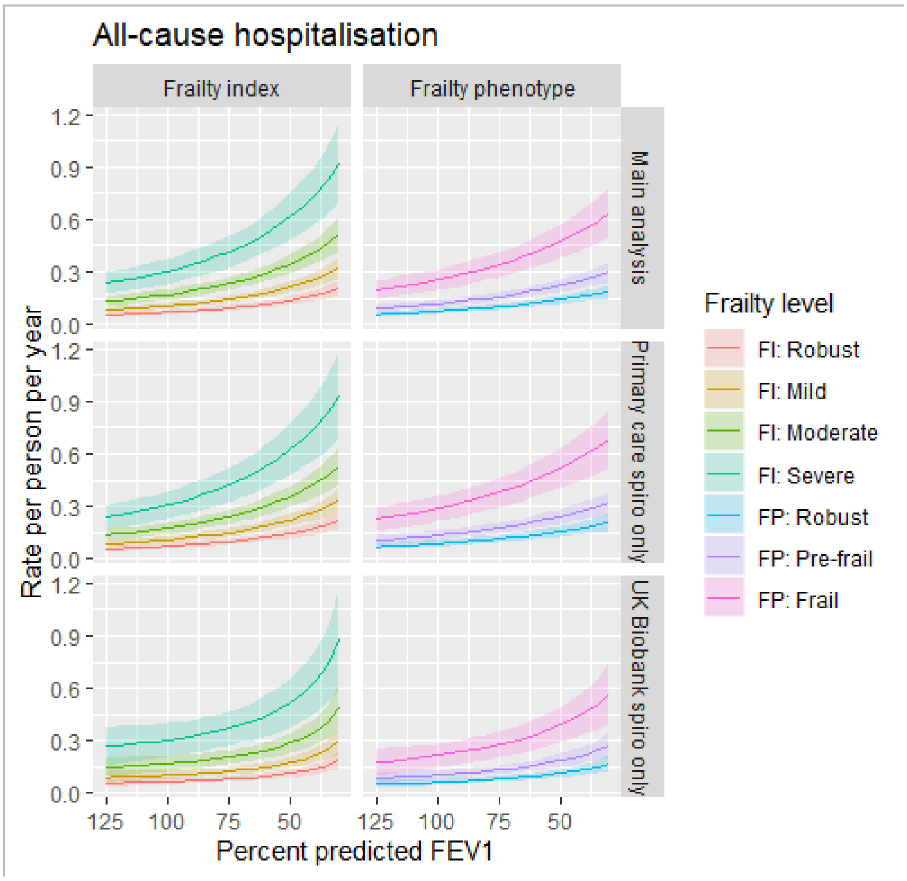
All-cause hospitalisation
Frailty index
Frailty phenotype
Main analysis
Primary care spiro only
UK Biobank spiro only
Rate per person per year
1.2
0.9
0.6
0.3
0.0
125
100
75
50
Percent predicted FEV1
Frailty level
FI: Robust
FI: Mild
FI: Moderate
FI: Severe
FP: Robust
FP: Pre-frail
FP: Frail

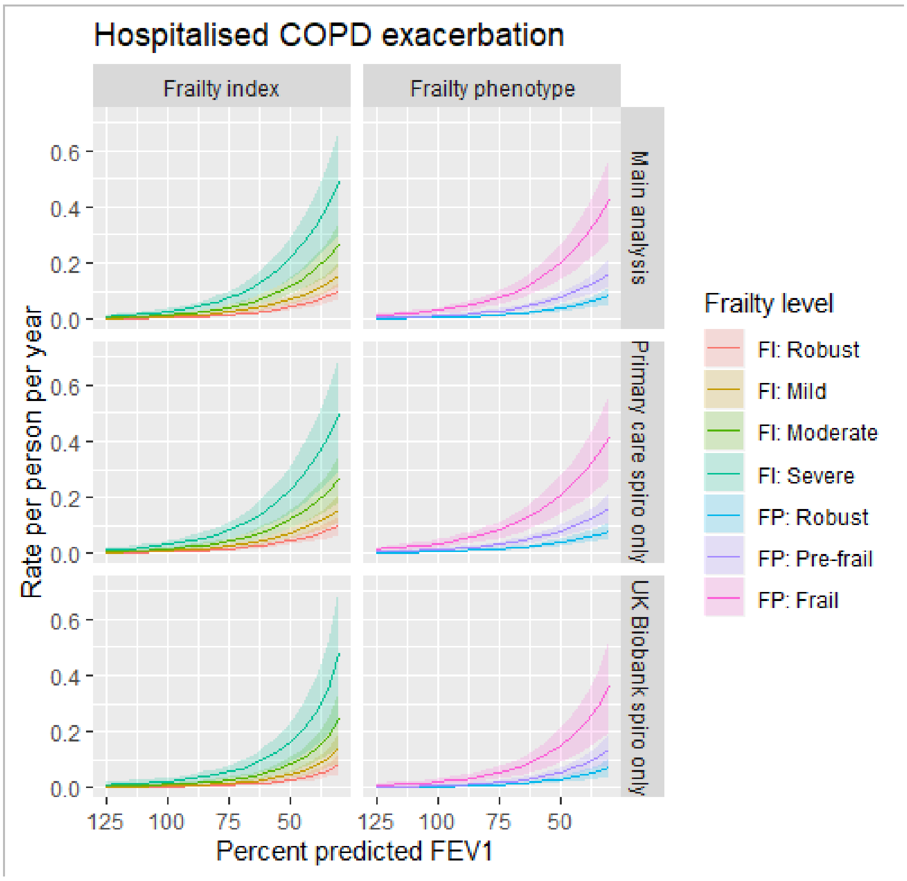
Hospitalised COPD exacerbation
Frailty index
Frailty phenotype
Main analysis
Primary care spiro only
UK Biobank spiro only
Rate per person per year
0.0
0.2
0.4
0.6
125
100
75
50
Percent predicted FEV1
Frailty level
FI: Robust
FI: Mild
FI: Moderate
FI: Severe
FP: Robust
FP: Pre-frail
FP: Frail

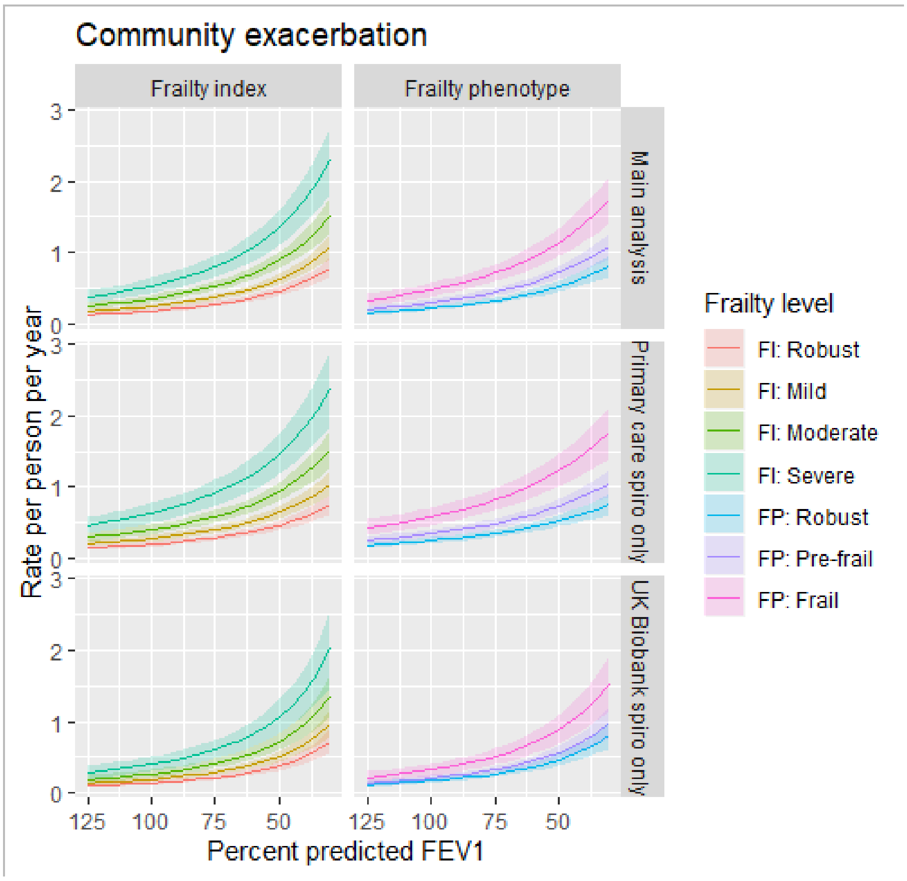
Community exacerbation
Frailty index
Frailty phenotype
Main analysis
Primary care spiro only
UK Biobank spiro only
Rate per person per year
Percent predicted FEV1
0
1
2
3
125
100
75
50
Frailty level
FI: Robust
FI: Mild
FI: Moderate
FI: Severe
FP: Robust
FP: Pre-frail
FP: Frail
